# Genetic diversity in Napier grass (*Pennisetum purpureum*) cultivars: implications for breeding and conservation

**DOI:** 10.1093/aobpla/plt022

**Published:** 2013-03-27

**Authors:** Bramwel W. Wanjala, Meshack Obonyo, Francis N. Wachira, Alice Muchugi, Margaret Mulaa, Jagger Harvey, Robert A. Skilton, Janice Proud, Jean Hanson

**Affiliations:** 1 Biotechnology and Biodiversity, Kenya Agricultural Research Institute, PO Box 14733, Nairobi 00800, Kenya; 2 Biochemistry and Molecular Biology Department, Egerton University, PO Box 563, Egerton 20115, Kenya; 3 Biosciences Eastern and Central Africa (BecA) at ILRI Hub, PO Box 30709, Nairobi 00100, Kenya; 4 International Centre for Research in Agroforestry, World Agroforestry Centre, United Nations Avenue, Gigiri, PO Box 30677, Nairobi 00100, Kenya; 5 Kenya Agricultural Research Institute, PO Box 450, Kitale 30200, Kenya; 6 International Livestock Research Institute (ILRI), PO Box 5689, Addis Ababa, Ethiopia; 7 Present address: Association for Strengthening Agricultural Research in Eastern and Central Africa, PO Box 765, Entebbe, Uganda

**Keywords:** AFLP, conservation, cultivars, genetic diversity, germplasm, Napier grass

## Abstract

Napier grass is an important forage crop for dairy production in the tropics; as such, its existing genetic diversity needs to be assessed for conservation. The current study assessed the genetic variation of Napier grass collections from selected regions in Eastern Africa and the International Livestock Research Institute Forage Germplasm-Ethiopia. The diversity of 281 cultivars was investigated using five selective amplified fragment length polymorphism (AFLP) markers and classical population genetic parameters analysed using various software. The number of bands generated was 216 with fragments per primer set ranging from 50 to 115. Mean percentage polymorphic loci was 63.40. Genetic diversity coefficients based on Nei's genetic diversity ranged from 0.0783 to 0.2142 and Shannon's information index ranged from 0.1293 to 0.3445. The *F*st value obtained was moderately significant (*F*st = 0.1688). Neighbour-joining analysis gave two distinct clusters which did not reflect geographical locations. Analysis of molecular variance showed all variance components to be highly significant (*P* < 0.001), indicating more variation within (91 %) than between populations (9 %). Results suggested moderate genetic differentiation among Napier grass populations sampled, which could imply a high germplasm exchange within the region. The AFLP markers used in this study efficiently discriminate among cultivars and could be useful in identification and germplasm conservation.

## Abstract (precis)

Napier grass is an important forage for smallholder dairy farms. However, there has been a comparatively low effort to improve Napier grass. It is necessary to strengthen forage breeding programs for development of cultivars with superior traits like. With a high rich gene pool; correct identification of Napier grass accessions is a prerequisite because the existing germplasm information is scanty and cannot be relied upon for crop improvement. Thus the genetic assessment of various Napier grass accessions from the Eastern Africa region is important for correct cultivar identification in order to fully exploit them in crop improvement strategies.

## Introduction

In East Africa, Napier grass (*Pennisetum purpureum*) is a perennial grass grown widely as a fodder crop and feed for the cut-and-carry zero-grazing dairy systems (Bayer 1990) and constitutes up to 80 % of forage for smallholder dairy farms (Staal *et al*. 1987). It is the forage of choice not only in the tropics but also worldwide (Hanna *et al*. 2004) due to its desirable traits such as tolerance to drought and a wide range of soil conditions, and high photosynthetic and water-use efficiency (Anderson *et al*. 2008).

While much attention has been directed towards research for improving the productivity of major cereal crops (Katz 2003), there has been comparatively little effort to improve Napier grass—an important forage crop that has been grown over centuries and currently enjoys a multiplicity of uses besides conventional animal consumption (Jaradat 2010). This is key among the drivers of renewed research interest in this otherwise previously neglected crop. However, the productivity of Napier grass is limited by several factors especially emerging diseases, mainly Napier grass stunt disease and Napier grass head smut disease, which constrains the growth of the smallholder dairy industry (New Agriculturists 2009). For this reason, it is necessary to strengthen forage breeding programmes for the development of disease-resistant cultivars (Boa *et al*. 2005).

Correct identification of Napier grass accessions is a prerequisite because the existing germplasm information is scanty and cannot be relied upon for crop improvement, since cultivar discrimination has predominantly relied on morphological and agronomic features and is the major cause of inconsistency in identification. Consequently, a number of Napier grass cultivars have been in circulation, often with more than one name (Struwig 2007).

Molecular markers have proven useful in distinguishing among morphologically related individuals within cultivars of the same plant species (Mohammadi and Prasanna 2003). Thus the genetic assessment of various Napier grass accessions from the Eastern Africa region is important for correct cultivar identification in order to exploit them fully in crop improvement strategies.

However, in genetic diversity studies, a wide range of marker systems require development in order to be used either singly or in combination, depending on the researcher's interest. Thus, most markers have some degree of shortcomings, which include: (i) restriction fragment length polymorphisms (RFLPs) are time consuming and involve expensive radioactive materials (Mondini *et al*. 2009), (ii) random amplified polymorphic DNAs (RAPDs) are irreproducible (Bardakci 2001), (iii) isozyme systems are few per species, (iv) amplified fragment length polymorphism (AFLP) band profiles cannot be interpreted in terms of loci, alleles and their dominance (Spooner *et al.* 2005), and (v) there is a lack of simple sequence repeats (SSRs) for Napier grass (Azevedo *et al*. 2012). Thus, due to a large number of loci analysed, high polymorphism levels, amenability to automation, high reproducibility without prior sequence knowledge and genome-wide marker distribution (Powell *et al*. 1996), the AFLP method (Vos *et al*. 1995) is considered more reliable and robust for an evaluation of genetic variability.

This study assessed the genetic variation between and within Napier grass collections comprising 281 accessions from selected regions in Eastern Africa (Kenya, Uganda, Tanzania and International Livestock Research Institute Forage Germplasm-Ethiopia). Implications of the findings for germplasm identification, breeding and conservation are discussed.

## Methods

### Source of cultivars

Cultivars were collected from selected regions in Kenya, Uganda and Tanzania between 2009 and 2010. Also included was a collection from the International Livestock Research Institute Forage Germplasm (ILRI-FG) which comprised cultivars from other parts of Africa, USA and accessions of unknown origin. The ILRI-FG was established in 1982 and has been maintained without external infusion (Table 1). Samples were selected based on morphological differences and collected from different regions with a range of ecological and altitudinal variations. They were then evaluated for nutritive parameters and biomass forage yield compared with the best local clone at 8 weeks post-harvest intervals. From the 281 clones, 3 young leaves without necrosis were collected and placed in a transparent polythene bag. Holes were punched to permit air flow. Samples were dried by placing them in a large plastic bag with silica. The silica gel was changed daily until samples were completely dry. The dried samples were then packaged in large polythene bags (without holes to prevent rehydration) and sent to the Biosciences east and central Africa (BecA)-ILRI Hub, Nairobi for analysis.

**Table 1:** Genetic diversity indices. Population, sub-population, sample size (*N*), mean diversity estimates (*H*) and Shannon's information index (*I*), polymorphic loci and % polymorphic loci. *Samples from Ethiopia = 2, Nigeria = 2 and Ghana = 2.

Populations	Sub-populations	*N*	*H*	*I*	No. of polymorphic loci	% Polymorphic loci
Kenya	Bungoma	27	0.1169	0.2076	167	77.3
	Busia	25	0.1197	0.2095	154	71.3
	Butere	22	0.1492	0.2569	187	86.6
	Alupe	11	0.1195	0.2071	140	64.8
	Mumias	35	0.1416	0.2464	193	89.4
Tanzania	Hai	5	0.1747	0.2675	116	53.7
	Lushoto	4	0.1727	0.2633	111	51.3
	Meru	3	0.1608	0.2390	92	42.6
	Muheza	14	0.1011	0.1771	128	59.3
	Tarime	19	0.1893	0.3113	186	83.1
Uganda	Kabarole	12	0.2130	0.3353	168	77.8
	Masaka	23	0.1244	0.2099	151	69.9
	Naro	5	0.0167	0.0266	13	32.5
	Soroti	16	0.2142	0.3445	189	87.5
ILRI-FG	Others	6*	0.0783	0.1293	71	32.9
	Swaziland	6	0.1576	0.2495	123	56.9
	Tanzania	5	0.1308	0.2035	93	43.1
	Unknown	14	0.1338	0.2258	145	67.1
	USA	16	0.1890	0.3044	170	78.7
	Zimbabwe-C	8	0.1844	0.2938	152	70.4

### DNA extraction

Genomic DNA was extracted following the cetyltrimethylammonium bromide (CTAB) method (Doyle and Doyle 1987) with some modifications. Dried leaves of ∼200–300 mg were cut into small pieces and transferred into 1.2-mL strip tubes containing two stainless steel grinding balls. Strip tubes were then cooled by immersing in liquid nitrogen. The frozen leaves were subsequently ground into fine powder using GenoGrinder-2000 at a speed of 500 strokes min^−1^ for 10 min. Strip tubes were centrifuged at 1000 rpm for 1 min to allow collection of the ground tissue at the bottom. Warm CTAB buffer (700 µL; 65 °C) (2 % CTAB, 100 mM Tris–HCl pH 8.0, 1.4 M NaCl, 20 mM EDTA, 2 % polyvinylpyrrolidone (PVP)) was added and the samples homogenized for 1 min by re-grinding in a GenoGrinder. Samples were incubated in a water bath at 65 °C for an hour with continuous shaking and mixing by inversion of strip tubes (every 15 min). Tubes were then removed from the water bath and left to cool at room temperature for 10 min, followed by centrifugation at 4000 rpm for 20 min. The aqueous layer was transferred into fresh strip tubes. Chloroform : isoamyl alcohol (24 : 1; 600 µL) was added and the contents mixed by gently inverting the tubes 10 times. Centrifugation was carried out at 4000 rpm for 20 min and the aqueous (top) layer transferred into a fresh strip tube. The above step was repeated twice and the aqueous layer transferred into fresh strip tubes. Ice-cold absolute ethanol : sodium acetate (25 : 1; 600 µL) was added gently and mixed by inversion, and a sample incubated at −20 °C for 45 min. DNA was pelleted by centrifugation at 4000 rpm for 20 min. The supernatant was removed; the pellet was washed with 500 µL of 70 % ethanol and left to stand for 5 min, then washed twice. The DNA pellet was air dried for 20 min, then dissolved in 100 µL of low-salt TE buffer (10 mM Tris–HCl pH 7.5, 1 mM EDTA). To free the DNA of RNA, 5 µL of ribonuclease A (RNase A), 10 mg/mL, were added and incubated at 37 °C for 30 min. DNA was re-precipitated by adding 200 µL of cold absolute ethanol and incubated at −20 °C for 30 min. DNA was pelleted by centrifuging at 4000 rpm for 20 min and washed with 70 % ethanol twice. The pellet was air dried for 30–60 min and resuspended in 100 µL of low-salt TE buffer.

### AFLP analysis

The AFLP protocol described by Vos *et al*. (1995) was employed. Sixty-four primer pairs were initially screened for their potential to produce scorable fragments (Applied Biosystems, Foster City, CA, USA). From 64 primer pairs, five were chosen based on their reproducibility and levels of fragment polymorphism (Table 2). Reproducibility was assessed by duplicating the same sample three times with different primer pair combinations to produce similar electropherograms. Polymerase chain reaction (PCR) products were prepared for separation on capillary electrophoresis (Applied Biosystems). Size separation of AFLP–PCR fragments was done on an ABI 3730 *xl* Genetic Analyzer (Applied Biosystems). Amplified fragment length polymorphism peaks from the Genetic Analyzer were sized and alleles scored with GeneMapper version 4.1 (Applied Biosystems AFLP Plant Mapping Protocol, 2005). Scored results were exported to an Excel matrix with values 1 (allele present) or 0 (allele absent).

**Table 2:** Selected selective AFLP primer combinations.

Primer no.	EcoRI primer	MseI primer	Dye	Colour
1	EcoRI + AAC	MseI + CAT	NED	Yellow
2	EcoRI + ACC	MseI + CAA	NED	Yellow
3	EcoRI + ACT	MseI + CAG	6-FAM	Blue
4	EcoRI + ACT	MseI + CAT	6-FAM	Blue
5	EcoRI + AGG	MseI + CAC	JOE	Green

### Data analyses

Genetic diversity, ordination analysis and analysis of molecular variance (AMOVA)—for diversity among and within populations—were determined using GenAlEx software (Peakall and Smouse 2009). PopGen32 (Yeh *et al*. 1997) was used for the population-based approach using *F* statistics, gene diversity over loci, proportion of polymorphic loci, Shannon index and gene frequency (Nei 1987; McDermott and McDonald 1993). In addition, the genetic distance between any two populations (Schneider *et al*. 2000) was computed using Tools for Population Genetic Analysis (TFPGA) software (Miller 1997). To show the relationships between 281 cultivars, principal co-ordinate analysis and unweighted pair group method with arithmetic mean (UPGMA) were generated using Darwin software (Perrier and Jacquemoud-Collet 2006). About 2000 bootstrap replicates were used to determine branch support in the consensus tree.

## Results

### Evaluation of genetic diversity

From the initial 64 primers screened, 22 were efficient at the inter-population level and sufficiently polymorphic to discriminate clones within populations. Intra-population polymorphism obtained with the selected primers gave alleles unique to each individual from the same population. Five primers that generated electropherograms with high relative fluorescent units/(peaks) without background noise were further selected for genotyping. Amplified fragment length polymorphism fragments ranged from 50 to 500 base pairs. Polymorphic bands ranged from 50 to 115 with an average of 43 bands per primer from a total of 216 bands generated, accounting for 64.80 % of polymorphic loci observed. The percentage of polymorphic fragments within sub-populations ranged from 32.5 for the National Agricultural Research Organization (NARO) to 89.4 % for Mumias. Following the same order, the genetic diversity coefficients based on Nei's genetic diversity ranged from 0.0783 to 0.2142 and Shannon's information index ranged from 0.1293 to 0.3445 (Table 1).

### Phenetic analysis

**Figure 1.**
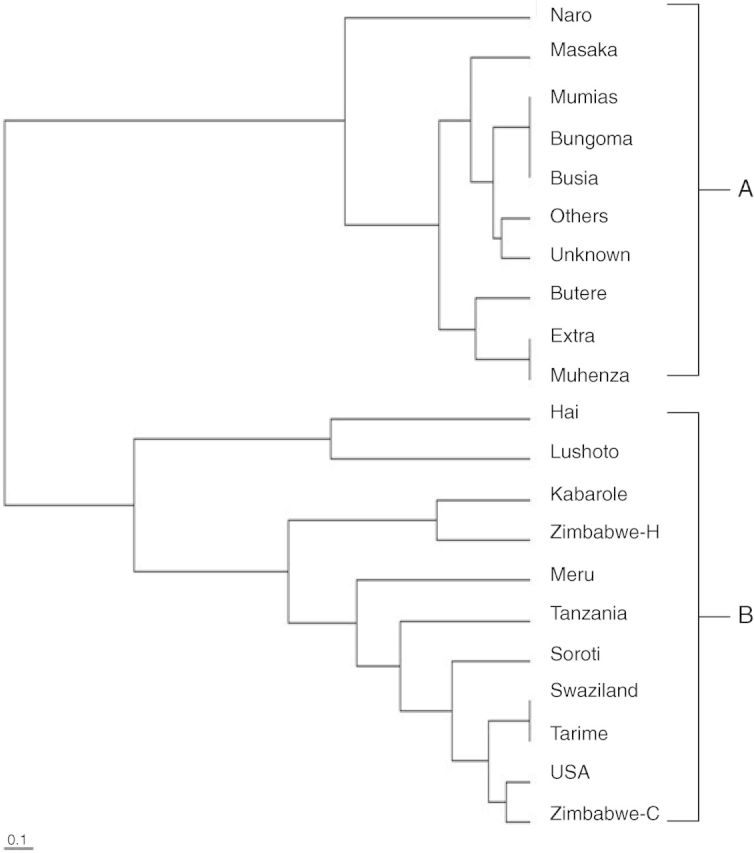
UPGMA dendrogram of 21 *P. purpureum* sub-populations from Kenya, Uganda, Tanzania and ILRI-FG using genetic distance (Nei 1979).

**Figure 2.**
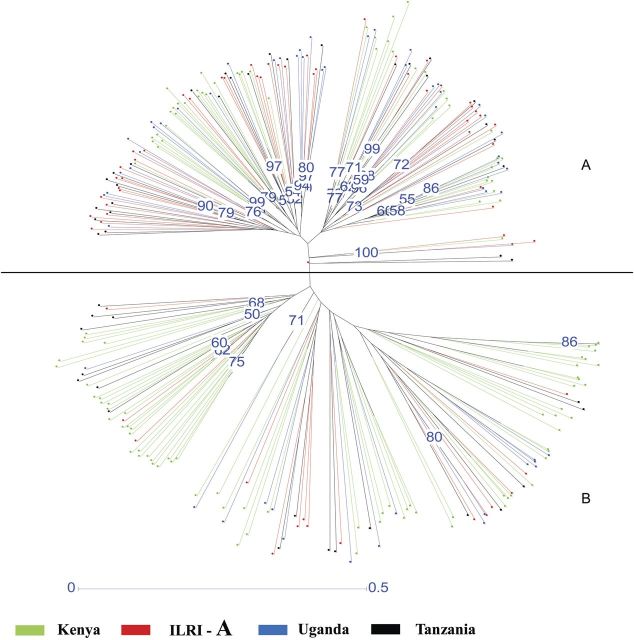
UPGMA neighbour-joining dendrogram of 281 Napier grass accessions computed from 216 polymorphic AFLP markers using Darwin hierarchical clustering with a bootstrap value at 2000.

The genetic distances between/among 21 Napier grass sub-populations (regions within countries) and four populations (countries: Kenya, Uganda, Tanzania and ILRI-FG) were subjected to hierarchical clustering by UPGMA (Table 3). This yielded two distinct clusters (A and B) which did not reflect the geographical locations of the 281 cultivars (Fig. 1). In addition, there was an overlap among cultivars spread across different clusters (Fig. 2). Pairwise comparison of genetic distance and similarity of populations revealed little genetic diversity within the Kenyan population and the ILRI-FG population, while it was moderate within the Uganda and Tanzanian populations.

**Table 3:** Nei's genetic distance of the 21 sub-populations of Napier grass based on AFLP analysis. Population codes: 1 = Bungoma, 2 = Busia, 3 = Butere, 4 = Extra, 5 = Hai, 6 = Kabarole, 7 = Lushoto, 8 = Masaka, 9 = Meru, 10 = Muhenza, 11 = Mumias, 12 = Naro, 13 = Others, 14 = Soroti, 15 = Swaziland, 16 = Tanzania, 17 = Tarime, 18 = Unknown, 19 = USA, 20 = Zimbabwe-cultivar and Zimbabwe-hybrids. Bold values are significant.

Pop ID	1	2	3	4	5	6	7	8	9	10	11	12	13	14	15	16	17	18	19	20	21
**1**	****																				
**2**	0.0006	****																			
**3**	0.0055	0.0034	****																		
**4**	0.0015	0.0003	0.0014	****																	
5	0.0531	0.0520	0.0487	0.0667	****																
**6**	0.0376	0.0372	0.0336	0.0458	0.0207	****															
**7**	0.0897	0.0855	0.0841	0.1024	0.0141	0.0384	****														
**8**	0.0032	0.0046	0.0142	0.0110	0.0419	0.0313	0.0794	****													
**9**	0.0151	0.0128	0.0132	0.0156	0.0334	0.0364	0.0614	0.0155	****												
**10**	0.0050	0.0033	0.0063	0.0002	0.0741	0.0541	0.1053	0.0151	0.0180	****											
**11**	**0.0001**	0.0006	0.0027	0.0021	0.0410	0.0302	0.0756	0.0030	0.0098	0.0057	****										
**12**	0.0108	0.0104	0.0239	0.0105	0.0971	0.0764	0.1338	0.0197	0.0261	0.0091	0.0156	****									
**13**	0.0018	0.0019	0.0136	0.0073	0.0571	0.0424	0.0884	0.0028	0.0134	0.0084	0.0040	0.0046	****								
**14**	0.0248	0.0240	0.0236	0.0329	0.0166	0.0080	0.0262	0.0210	0.0180	0.0360	0.0189	0.0533	0.0261	****							
**15**	0.0214	0.0205	0.0221	0.0307	0.0129	0.0114	0.0419	0.0132	0.0101	0.0379	0.0147	0.0502	0.0207	0.0058	****						
**16**	0.0252	0.0243	0.0323	0.0358	0.0223	0.0140	0.0423	0.0159	0.0245	0.0399	0.0207	0.0482	0.0180	0.0091	0.0045	****					
**17**	0.0151	0.0142	0.0162	0.0220	0.0126	0.0119	0.0382	0.0103	0.0072	0.0255	0.0095	0.0399	0.0138	0.0049	0.0020	0.0049	****				
**18**	0.0041	0.0014	0.0061	0.0046	0.0425	0.0313	0.0639	0.0072	0.0080	0.0059	0.0023	0.0130	0.0021	0.0165	0.0152	0.0151	0.0087	****			
**19**	0.0276	0.0262	0.0250	0.0349	0.0088	0.0159	0.0230	0.0228	0.0112	0.0391	0.0200	0.0536	0.0257	0.0036	0.0036	0.0080	0.0027	0.0148	****		
**20**	0.0237	0.0219	0.0153	0.0301	0.0130	0.0163	0.0343	0.0216	0.0026	0.0345	0.0148	0.0554	0.0280	0.0078	0.0020	0.0194	0.0033	0.0160	0.0017	****	
**21**	0.0492	0.0487	0.0523	0.0645	0.0201	0.0066	0.0392	0.0349	0.0430	0.0707	0.0420	0.0802	0.0431	0.0080	0.0094	0.0100	0.0165	0.0387	0.0157	0.0231	****

### Population structure among and within populations

Variance components obtained by AMOVA were highly significant (*P* < 0.001) with more variation within (91 %) than between populations (9 %) (Table 4). Similarly, nested analysis partitioned by country, within population, Uganda 83 %, Kenya 97 %, Tanzania 86 % and ILRI-FG 96 %, was also highly significant (*P* < 0.001). On the other hand, variation among population showed the Kenyan population as having the least variation (3 %) while the most variation was among the Ugandan population (17 %).

**Table 4:** Analysis of molecular variance for 281 genotypes from 21 Napier grass populations based on 216 AFLP markers. Df, degrees of freedom; Ss, sum of squares; Ms, mean square; Est.var, estimated variation.

Source of variation	Df	Ss	Ms	Est.var	Percentage	*P* value
All populations						
Among population	20	1125.94	56.29	2.46	9	0.001
Within population	260	6249.76	24.04	24.04	91	0.001
Kenya population						
Among population	4	105.08	26.27	0.15	3	0.001
Within population	115	2611.36	22.71	22.71	97	0.001
Tanzania population						
Among population	4	231.73	57.93	4.06	14	0.001
Within population	40	1038.36	25.959	25.96	86	0.001
Uganda population						
Among population	3	273.893	91.298	5.15	17	0.001
Within population	52	1269.55	24.414	24.41	83	0.001
ILRI-FG						
Among population	6	3.018	0.503	0.001	4	0.001
Within population	53	26.10	0.492	0.49	96	0.001

## Discussion

### Reliability of AFLP markers

A good molecular marker must separate individuals' inter-populations and still be sufficiently polymorphic at the intra-population level to precisely identify clones (Mueller and Wolfenbarger 1999). Amplified fragment length polymorphism employed in the current study generated numerous highly polymorphic alleles, which corroborates the findings of Vos *et al*. (1995) that a large number of alleles are important for accurately estimating the genetic diversity of a germplasm. While microsatellites are among the most commonly used markers due to their locus specificity, co-dominant nature, high polymorphism and reproducibility, their development and application have been restricted to a few agriculturally important crops since they first require identification via genome sequencing (Powell *et al*. 1996). The genome of Napier grass has not been sequenced and the inputs involved are high and limit the development of microsatellite for this crop. In addition, Napier grass is a tetraploid (2*n* = 4*x* = 28) and triploid and hexaploid hybrids occur between it and pearl millet (Techio *et al*. 2010). This presents a challenge in establishing microsatellites that can clearly discriminate the different ploidy levels. The multi-locus nature of AFLP thus makes it reliable over the microsatellite procedure as it scans the entire genome.

The resolving power of a marker is an important index in selecting the most informative markers for diversity studies (Azevedo *et al*. 2012). For example, Harris *et al*. (2009) recorded a resolving power of 13.2 for AFLP while Azevedo *et al*. (2012) found a resolving power of 1.55 for microsatellites, implying that AFLP is more informative. The current study employed five primer pair combinations which were found to be polymorphic and sufficient in discriminating Napier grass cultivars. This is in agreement with the findings of Ellis *et al*. (1997) that by choosing the six best combinations of primers it would be possible to explain over 80 % of expected species relatedness; in addition Saunders *et al*. (2001) demonstrated that differences in AFLP-DNA fragments would be detected when no more than 3–7 primer pairs are used. Thus, this study established a reliable AFLP procedure for Napier grass.

### Evaluation of genetic diversity

The current study was founded on the premise that Napier grass accessions could segregate based on geographical origin and hence would offer a unique genetic resource for breeding programmes in the region. However, the converse appears true as there is moderate diversity among accessions in the region. The clustering suggests genotype overlap and redundancy possibly due to low gene flow. The materials under circulation share ancestry especially since Napier grass is clonally propagated, the users may be sharing planting materials. These findings contradict previous studies that demonstrated clustering of Napier grass accessions based on geographical location (Lowe *et al*. 2003; Harris *et al*. 2009). Despite the fact that Harris *et al*. (2009) used AFLP markers, different primer pair combinations and samples were analysed. On the other hand, Lowe *et al*. used different samples from those used in this study. In addition, the marker employed was RAPDS, which has several limitations, among them lack of reproducibility The low genetic distance observed among the Kenyan sub-populations may be attributed to the close proximity of the sampled areas (Bungoma, Extra-Alupe, Busia and Mumias), which are in a radius of ∼60 km. Similarly, this applies to ILRI-FG, Tanzania and Uganda populations, which had moderate genetic distances. The ‘Tanzania’ sub-population, from ILRI-FG, clustered with Tanzanian populations, Lushoto, Tarime and Meru, indicating ancestry from Tanzania. The sub-ILRI-FG germplasm is diverse with most originating from Zimbabwe and the USA; however, their origin could not be ascertained. The USA is physically isolated from Africa, but these individuals were still flanked by the cultivars collected from Africa. This could suggest that they had not yet diversified from their African relatives. Struwig (2007) using AFLP markers on ILRI-FG genotypes observed that cultivars did not cluster based on geographical origin, which is consistent with observations of the current study.

### Population genetic structure

Genetic structure is affected by several factors including breeding systems, genetic drift, population age and size, environmental heterogeneity, seed dispersal, gene flow, evolutionary history as well as natural selection (Hamrick and Godt 1990). This is probably attributable to the out-crossing nature of Napier grass, which has higher levels of genetic diversity and lower differentiation among populations than in selfing and clonal plants (Rossetto *et al*. 1995). Similar results were observed in other out-crossing species such as perennial rye grass (*Lolium perenne*), meadow fescue (*Festuca pratensis*), orchard grass (*Dactylis glomerata*) and Rhodes grass (*Chloris gayana*) (Huff 1997; Kölliker *et al*. 1998; Ubi *et al*. 2003).

The *F*st values obtained between the 21 sub-populations indicate moderate genetic differentiation in Napier grass cultivars (Hartl and Clark 1989). Since the grass is a clonal plant with low seed setting and germination, it is spread by asexual stem reproduction. Therefore, gene flow among cultivars is low and most genetic variation resides between rather than within cultivars (Xie *et al*. 2009). Another probable reason for high within-population variation may be caused by Napier grass being a highly heterozygous tetraploid species. Variations in Napier grass cultivars are expected to be high due to its rich gene pool and wide parental diversity (Azevedo *et al*. 2012). Taxonomic representation is based on the cross-ability of wild species and the domesticated form and the amount of gene flow occurring (Robert *et al*. 1991). The genus *Pennisetum* has three gene pools. The primary gene pool occurs between the domestication of *Pennisetum glaucum* and wild weedy forms of *P. glaucum*. The secondary gene pool is between perennial and wild relative (*P. purpureum* to *P. glaucum)*. They cross easily but their hybrids are sterile. The tertiary gene pool comprises true biological species compared with the primary and secondary gene pools (Robert *et al*. 1991). However, strong reproductive barriers impede natural gene flow and the occurrence of hybrids between tertiary versus other forms (Robert *et al*. 1991). Therefore, higher levels of genetic differentiation among cultivars could arise from the pressure of artificial selection, which occurs rarely.

### Implications for conservation and improvement

Africa is believed to be the centre for domestication of Napier grass (Azevedo *et al*. 2012) as it houses a majority of the *Pennisetum* gene pools (Techio *et al*. 2010). Thus, maintaining genetic diversity within natural populations can maximize their potential to withstand and adapt to biotic and abiotic pressures (Jump *et al*. 2009). The possibility of interspecific combination is important for breeding as it allows the transfer of alleles into species of agronomic importance, as has been done successfully between millet and other species of *Pennisetum* (Techio *et al*. 2010).

Assessment of the entire *Pennisetum* gene pool would make material available for breeding programmes without causing genetic erosion or loss of varieties. Thus there ought to be renewed efforts among researchers to populate the forage germplasm (ILRI-FG) with materials from different agroecological zones of the world. Potential sources of Napier grass diversity are South Africa, Brazil, Puerto Rico, the USA, Australia, China, Pakistan and India (Azevedo *et al*. 2012).

## Conclusions

The AFLP methodology developed in this study was able to discriminate among the Napier grass accessions and could be useful in screening cultivars.

## Sources of Funding

This work was supported by a grant (Napier grass smut and stunt resistance ASARECA project 06/RC01-FC-2-02) through the Association for Strengthening Agricultural Research in Eastern and Central Africa (ASARECA).

## Contributions by the Authors

B.W.W. conducted the lab work, and drafted and revised the manuscript with contributions from co-authors. M.O., F.N.W., J. Harvey and R.A.S. were primarily responsible for supervising the laboratory work and revising the manuscript. A.M. provided technical backstopping in the AFLP methodology and data analysis. M.M. was responsible for the field sampling of the Kenyan cultivars. J.P. coordinated all the activities of the project. J. Hanson conceived the study and obtained funding for it. All authors read and approved the final manuscript.

## Conflicts of Interest Statement

None declared.
